# Clinical application of a population-based input function (PBIF) for a shortened dynamic whole-body FDG-PET/CT protocol in patients with metastatic melanoma treated by immunotherapy

**DOI:** 10.1186/s40658-023-00601-3

**Published:** 2023-12-08

**Authors:** Mathieu Pavoine, Philippe Thuillier, Nicolas Karakatsanis, Delphine Legoupil, Karim Amrane, Romain Floch, Romain Le Pennec, Pierre-Yves Salaün, Ronan Abgral, David Bourhis

**Affiliations:** 1 Department of Nuclear Medicine, University Hospital, 2 Avenue Foch, 29200 Brest, France; 2 UMR INSERM 1304 GETBO, Brest, France; 3 Department of Endocrinology, University Hospital, Brest, France; 4 Department of Radiology, Weil Cornell Medical College of Cornell University, New York, NY USA; 5 Department of Dermatology, University Hospital, Brest, France; 6 Department of Oncology, Regional Hospital, Morlaix, France

**Keywords:** ^18^F-FDG, Dynamic whole-body PET, PBIF, Parametric imaging

## Abstract

**Background:**

The aim was to investigate the feasibility of a shortened dynamic whole-body (dWB) FDG-PET/CT protocol and Patlak imaging using a population-based input function (PBIF), instead of an image-derived input function (IDIF) across the 60-min post-injection period, and study its effect on the FDG influx rate (Ki) quantification in patients with metastatic melanoma (MM) undergoing immunotherapy.

**Methods:**

Thirty-seven patients were enrolled, including a PBIF modeling group (*n* = 17) and an independent validation cohort (*n* = 20) of MM from the ongoing prospective IMMUNOPET2 trial. All dWB-PET data were acquired on Vision 600 PET/CT systems. The PBIF was fitted using a Feng’s 4-compartments model and scaled to the individual IDIF tail’s section within the shortened acquisition time. The area under the curve (AUC) of PBIFs was compared to respective IDIFs AUC within 9 shortened time windows (TW) in terms of linear correlation (*R*^2^) and Bland–Altman tests. Ki metrics calculated with PBIF vs IDIF on 8 organs with physiological tracer uptake, 44 tumoral lesions of MM and 11 immune-induced inflammatory sites of pseudo-progression disease were also compared (Mann–Whitney test).

**Results:**

The mean ± SD relative AUC bias was calculated at 0.5 ± 3.8% (*R*^2^ = 0.961, AUC_PBIF_ = 1.007 × AUC_IDIF_). In terms of optimal use in routine practice and statistical results, the 5th–7th pass (*R*^2^ = 0.999 for both Ki mean and Ki max) and 5th–8th pass (mean ± SD bias = − 4.9 ± 6.5% for Ki mean and − 4.8% ± 5.6% for Ki max) windows were selected. There was no significant difference in Ki values from PBIF_5_7_ vs IDIF_5_7_ for physiological uptakes (*p* > 0.05) as well as for tumor lesions (mean ± SD Ki IDIF_5_7_ 3.07 ± 3.27 vs Ki PBIF_5_7_ 2.86 ± 2.96 100ml/ml/min, *p* = 0.586) and for inflammatory sites (mean ± SD Ki IDIF_5_7_ 1.13 ± 0.59 vs Ki PBIF_5_7_ 1.13 ± 0.55 100ml/ml/min, p = 0.98).

**Conclusion:**

Our study showed the feasibility of a shortened dWB-PET imaging protocol with a PBIF approach, allowing to reduce acquisition duration from 70 to 20 min with reasonable bias. These findings open perspectives for its clinical use in routine practice such as treatment response assessment in oncology.

**Supplementary Information:**

The online version contains supplementary material available at 10.1186/s40658-023-00601-3.

## Introduction

The major advance in cancer treatment over the past decade has undoubtedly been the introduction of immunotherapies with checkpoint inhibitor (ICIs). These treatments are currently indicated in metastatic melanoma (MM) showing an efficacy of about 40% and improving the 5-year overall survival (OS) of patients by 10 to 50% [1, 2]. Nevertheless, assessing the response to ICIs remains problematic with medical imaging in some circumstances. Indeed, the concept of pseudo-progression (PP) defined as a decrease or a stability in lesion size and metabolism after initially simulating a progression is now recognized [3]. Thus, this phenomenon of PP is the consequence of a significant peri-tumor inflammatory reactivity following the initiation of treatment that can falsely simulate a morpho-metabolic progression disease (PD). It occurs in 4–12% of MM treated with ICIs depending on the series [4, 5].

18Fluoro-desoxyglucose positron emission (FDG-PET/CT) is a functional imaging technique that can be proposed for assessing response of MM to systemic treatments, according to standard guidelines [6, 7]. The usual PET interpretation criteria have originally been developed to assess therapeutic response to chemotherapy, in analyzing variation of standardized uptake values (SUV) metrics between two scans [8, 9]. Therefore, in this area of ICIs, a new notion of unconfirmed progression (UP) had to be introduced instead of PD with the uncertainty related to PP. So, in case of UP, early re-assessment by FDG-PET/CT after 1 or 2 new cycles of treatment should be performed to confirm or exclude progression disease [10, 11]. With such a background and with the known limitations of the SUV metric [12, 13], finding ways to diagnose pseudo-progression early and accurately is a real challenge in FDG-PET/CT.

Dynamic whole-body (dWB) PET acquisition [14, 15] is an innovative method to assess the spatio-temporal distribution of the administered radiotracer in the whole body (WB), allowing at each voxel the direct calculation of the macro-kinetic tracer parameters, such as the tracer uptake rate (Ki), using robust Patlak graphical analysis [16]. The resulting Ki parametric images have been reported to aid in lesion detection and characterization of oncologic diseases compared to the standard SUV metrics alone [17, 18]. Indeed, few studies have shown a significant difference between the mean Ki values of inflammatory cells and tumor processes, which could provide a solution to differentiate PP and PD with WB dyn FDG-PET/CT [19–21]. Nevertheless, dWB-PET acquisition remains a long (around 60 min) and complex procedure requiring optimizations to facilitate its use in clinical routine [22, 23]. First, the use of an image-derived input function (IDIF) has been proposed as a less invasive method than arterial blood sampling to estimate blood IF, required input to model time–activity curves in physiological or tumor tissues [24–26]. Next, modeling a population-based input function (PBIF) was recently investigated to overcome the main time-consuming problem of this WB multi-passes PET procedure [27–29]. To date, few studies have already clinically validated a shortened duration WB FDG-PET/CT Patlak imaging using such PBIF [30–33].

IMMUNO-PET2 (NTC 04272658) is an ongoing single-center prospective observational trial investigating the diagnostic value of Ki metrics in dWB FDG-PET/CT to differentiate PP to PD of metastatic melanoma under ICI.

The aims of this ancillary study were: (i) to develop a population-based FDG input function (PBIF) model using an independent control group of patients, (ii) to demonstrate the clinical feasibility of a shortened dWB FDG-PET/CT examination protocol using the above PBIF model in the first patients included in this trial and (iii) to validate the WB Ki images quantification, measured on voxelwise direct parametric reconstructions, as estimated with PBIF from a reduced number of WB-passes, against the more accurate estimation of Ki with IDIF where all passes acquired during the first 60min post-injection were utilized.

## Material and methods

### Population

This is an ancillary study of IMMUNO-PET2 trial, an ongoing single-center prospective observational cohort study (NTC 04272658) exploring dynamic whole-body (dWB) FDG-PET/CT role in management of patients ≥ 18 years old, with stage IV metastatic melanoma (MM) treated with immunotherapies with checkpoint inhibitor (ICI).

The protocol was approved by the institutional medical ethics committee of Brest (29BRC19.0194). Informed consent was obtained from all the patients to participate in the study.

A total of 37 patients were recruited. The subjects were divided into two groups: a PBIF modeling group (*n* = 17) composed of healthy volunteers (training group G1) and an independent cohort of patients (*n* = 20) with MM (validation group G2).

### PET/CT system

All dynamic FDG-PET/CT acquisitions were performed on two digital Biograph Vision 600 systems (Siemens©, Erlangen, Germany) with similar following settings:

- CT data were acquired after injection of intravenous iodine contrast agent (1.5 mL.kg-1), unless contraindicated. The CT consisted of a 64-slice multidetector-row spiral scanner with a transverse field of view of 500 mm. The CT parameters were: collimation of 16 × 1.2mm, pitch = 1, tube voltage and exposure automatically regulated (CarekV, CareDose 4D) with 120kV and 80 Qref mAs as basic parameters. The CT images were reconstructed with an iterative method (SAFIRE, strength 5).
- PET data were reconstructed using an iterative reconstruction algorithm (OSEM 3D, 3 iterations and 5 subsets), with “time of flight” (ToF) and point-spread-function (PSF) correction (TrueX). PET images were corrected for random coincidence (DLYD), scatter (Model based) and attenuation using CT data. Gaussian filter (FWHM = 2 mm) was applied. The size of the transaxial reconstruction was 440 × 440 (voxel size = 1.65 × 1.65 × 1.65 mm).

### Whole-body PET protocol

PET images were acquired immediately after a manual injection of approximately 3 MBq/kg of FDG. The dWB-PET acquisition was performed in two steps according to the methodology previously described by Karakatsanis et al. [14, 17, 22].

A single-bed dynamic acquisition centered on the cardiac area (dCB) was followed by a whole-body (including lower limbs) dynamic acquisition in continuous bed motion, with a total duration of approximately 70 min: 6-min dCB (12 images × 5s, 6 images × 10s, 8 images × 30s) + WBdyn acquisition (8 passes of approximately 8 min/pass, performed with a constant table speed of 4 mm.s^−1^). During each of these passes, the PET data acquired over the cardiac region were used to complete the input function.

### Parametric Patlak imaging

Patlak reconstructions were performed using a direct 4D nested Patlak expectation–maximization reconstruction in the validation group, using both IDIFs and the scaled PBIFs to obtain parametric images [23, 34].

The Patlak method [16] is a graphical method to characterize the administered tracer’s macro-kinetic features based on the 3-compartment model. It consists in plotting the tracer concentration in a region of interest (ROI) as a function of post-injection mid-frame time points t, *C*_T_(*t*), divided by the concentration in the blood pool across the same time frames, *C*_B_(*t*), against the time integral of *C*_B_(*t*) between the injection time (*t* = 0) and the mid-frame times *t*, divided by *C*_B_(*t*). This model is described by Eq. 1:

1 $$\frac{{C}_{\mathrm{T}}\left(t\right)}{{C}_{\mathrm{B}}\left(t\right)}= {K}_{i} \frac{{\int }_{0}^{t}{C}_{\mathrm{B}}\left(\tau \right)\mathrm{d}\tau }{{C}_{\mathrm{B}}\left(t\right)}+ {V}_{b}$$

The slope *K*_i_ (Ki) represents the net influx of the tracer in min^−1^ and the intercept *V*_b_ (Vb) the total blood distribution volume ratio in the region of interest (ROI) or a voxel, in %. The Patlak graphical analysis involves the linear fit of the measured *C*_T_(*t*) and *C*_B_(*t*) PET data to Eq. (1) to estimate the slope Ki and intercept Vb parameters at each ROI or voxel.

### Input function

Theoretically and as a gold standard, an arterial blood sample is required to obtain an IF. However, it remains a complicated invasive method to implement in clinical routine. As previously described to overcome this problem [24–26], our digital PET system allowed to quantify total radioactivity concentration in the whole blood for extracting an IDIF and later for building the PBIF model. A spherical 1-cm^3^ VOI was automatically generated in the left ventricle and away from the myocardium to mitigate any partial volume effects [35] on CT corresponding to dCB and dWB-PET acquisitions by using an ALPHA algorithm (Automated Landmarking and Parsing of Human Anatomy), Siemens-Healthineers, PET-CT system VB80 [36]. During the first 25 min, a piece-wise linear fit was applied between every frame, and then, a decreasing exponential fit was applied (Eq. 2):

2 $$A={e}^{-\lambda .t}$$

where* A* is the intercept and *λ* is the rate constant of the exponential*.*

#### PBIF creation and IDIF validation cohort

##### PBIF modeling and fitting (G1)

The first step was to model a PBIF from the 17 IDIFs collected in the training group G1. IFs were resampled using liner interpolation (Matlab), in order to match the time steps between each of them all along the dynWB PET datasets.

The second step was to synchronize each IF on the shortest time to peak (TtP) to obtain an average function as already described by Wu et al. [37]. Then, a Feng’s 4-compartment model [38], describing the time–activity curve of tracer in the plasma, was finally applied to this average curve as follows (Eq. 3):

3 $${\text{SUV}}\left( t \right) = \left\{ {\begin{array}{*{20}c} 0 & {{\text{if }}t < \tau } \\ {\left[ {A_{1} \left( {t - \tau } \right) - A_{2} - A_{3} } \right]e^{{ - \lambda _{1} \left( {t - \tau } \right)}} + A_{2} e^{{ - \lambda _{2} (t - \tau )}} + A_{3} e^{{ - \lambda _{3} (t - \tau )}} } & {{\text{if }}t \ge \tau } \\ \end{array} } \right.$$

where *λ1, λ2, λ3* are the eigenvalues of the model*; A1, A2, A3* the coefficient constants*; τ* the time delay constant (in min).

To obtain the Feng’s model parameter from discrete data, we used the software SciDAVis©. The fitting is done by minimizing the least-square difference between the data points and the Y values of the function. To ensure that the calculation converged on a satisfactory solution, we manually entered the initial guesses and a constant time delay τ (min). A Levenberg–Marquardt algorithm is used to solve the nonlinear least-square problems.

##### Validation cohort (G2)

The third step was to validate the modeling PBIF in the validation group G2, including 20 patients with metastatic melanoma. Due to software limitation, the PBIF was scaled to all disposable points of the tail part of IDIF, generated from the late whole-body dynamic acquisition (from 1st to 8th passes, 11–70 min). This scaled PBIF was used to reconstruct different parametric images, using different time windows (TW) for the Patlak parameters calculation (Ki, VD) as follows: 2nd-4th or 2_4 (20–35 min), 3rd-5th or 3_5 (28–45 min), 4th-6th or 4_6 (35–53 min), 5th-7th or 5_7 (45–61 min), 2nd-5th or 2_5 (20–45 min), 3rd-6th or 3_6 (28–53 min), 4th-7th or 4_7 (35–61 min), 5th-8th or 5_8 (45–70 min), 2nd-7th or 2_7 (20–61 min).

#### PET quantitative analysis

A spherical 2-cm-diameter ROI was drawn, by the same operator, on the gold-standard SUV images, over an uninvaded part of 8 different organs (brain, muscle, aorta, myocardium, lung, liver, spleen, bone), and a spherical VOI was drawn over a maximum of 5 melanoma metastasis per patient using an advanced gradient-based segmentation tool (PET-Edge + ®) [39]. These ROIs were finally fused to the different reconstructed parametric images, Ki and Vb, to generate mean and max physiological and tumoral values (in 100ml/ml/min and %).

### Statistical analysis

The accuracy of scaled PBIFs was assessed by comparing their area under the curve (AUC) against that of the respective individual IDIFs. AUC comparisons were performed using both a linear correlation test (*R*^2^ and slope) and a Bland–Altman test (mean bias; confidence interval 95%). The physiological and pathological Ki values calculated using the scaled PBIFs were also compared against those estimated with the respective IDIF using a Mann–Whitney test.

All statistical analyses were performed using XLStat 2022 (Addinsoft©, Paris, France) and Excel (Microsoft©, Redmond, Washington, USA) softwares.

## Results

### Characteristics of the cohort

Demographic data of both training and validation groups are listed in Table 1.

**Table 1:** Patient characteristics

Parameters	Training group G1 (*n* = 17)	Validation group G2 (*n* = 20)
Gender** (M/F)**	7/10	13/7
Age (years old; mean ± SD)	62 ± 13	68 ± 14
BMI (kg/m^2^; mean ± SD)	25 ± 5	27 ± 5
**Activity (MBq; mean ± SD)**	207 ± 40	221 ± 37

### PBIF creation

The final parameters describing the PBIF by Feng’s model that best-fitted the average IDIFs curve are summarized in Table 2.

**Table 2:** PBIF parameter estimates (Feng's model)

Parameters	Values
A1	166.95 ± 1.33
A2	4.18 ± 0.01
A3	3.33 ± 0,01
λ1 (min^−1^)	4.66 ± 0,02
λ2 (min^−1^)	0.16 ± 1E-3
λ3 (min^−1^)	0.008 ± 5E-5
τ (min)	0.3

**Fig. 1:**
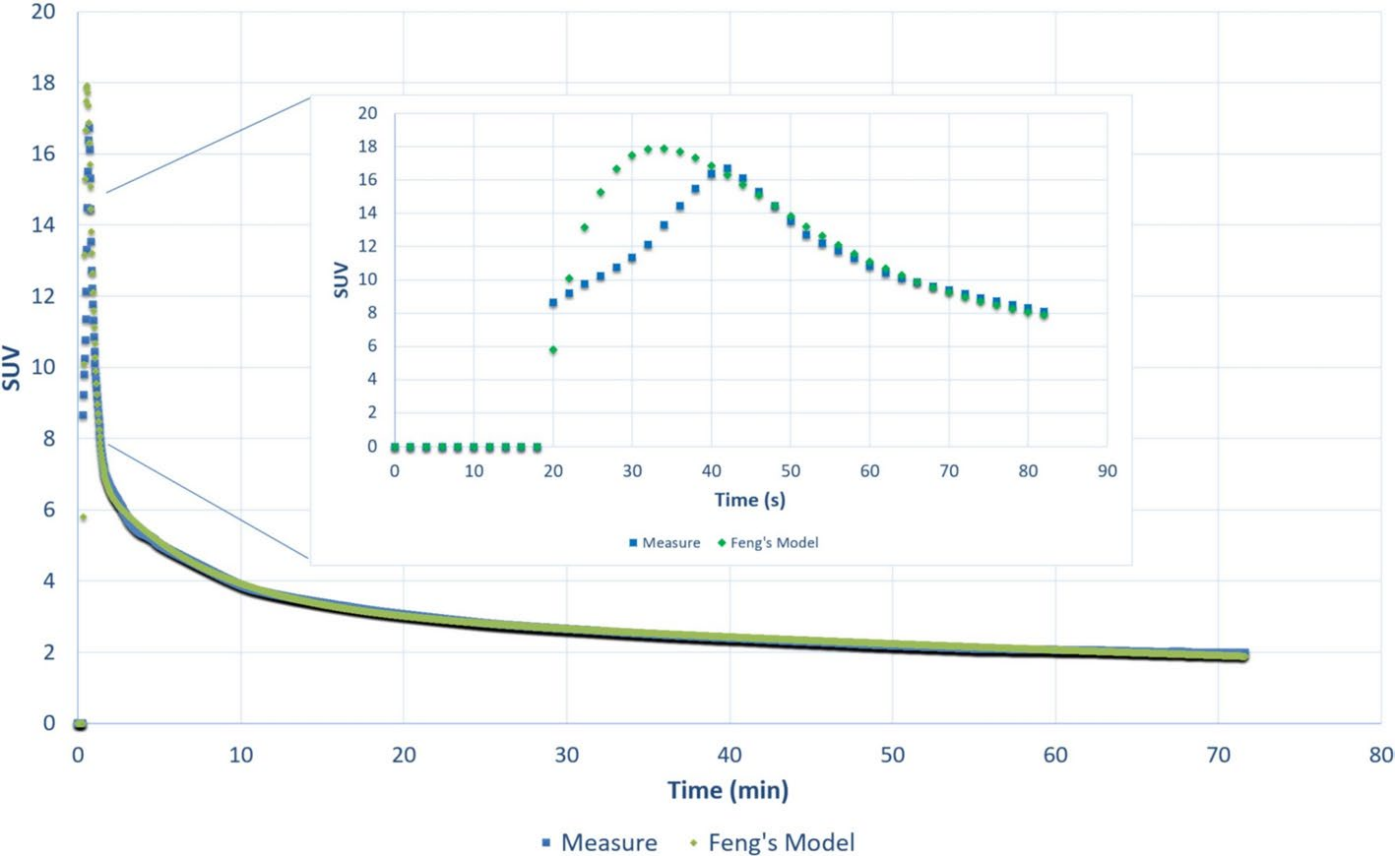
Comparison of the average IDIFs curve from each patient of the training group G1 (*n* = 17) and the corresponding PBIF (Feng's model). Zoom on the first 90 s

The average curve of the IDIFs from the training group G1 and the associated PBIF Feng’s model is shown in Fig. 1.

The correlation coefficient between the model and the average training points was calculated at *R*^2^ = 0.998, and the root-mean-square error (rmse) was 0.30. The most significant differences were found before the FDG absorption peak during the first 20 s without impact on the overall deviation estimated at 0.8%. 

### Comparison of AUC_PBIF_ and AUC_IDIF_

**Fig. 2:**
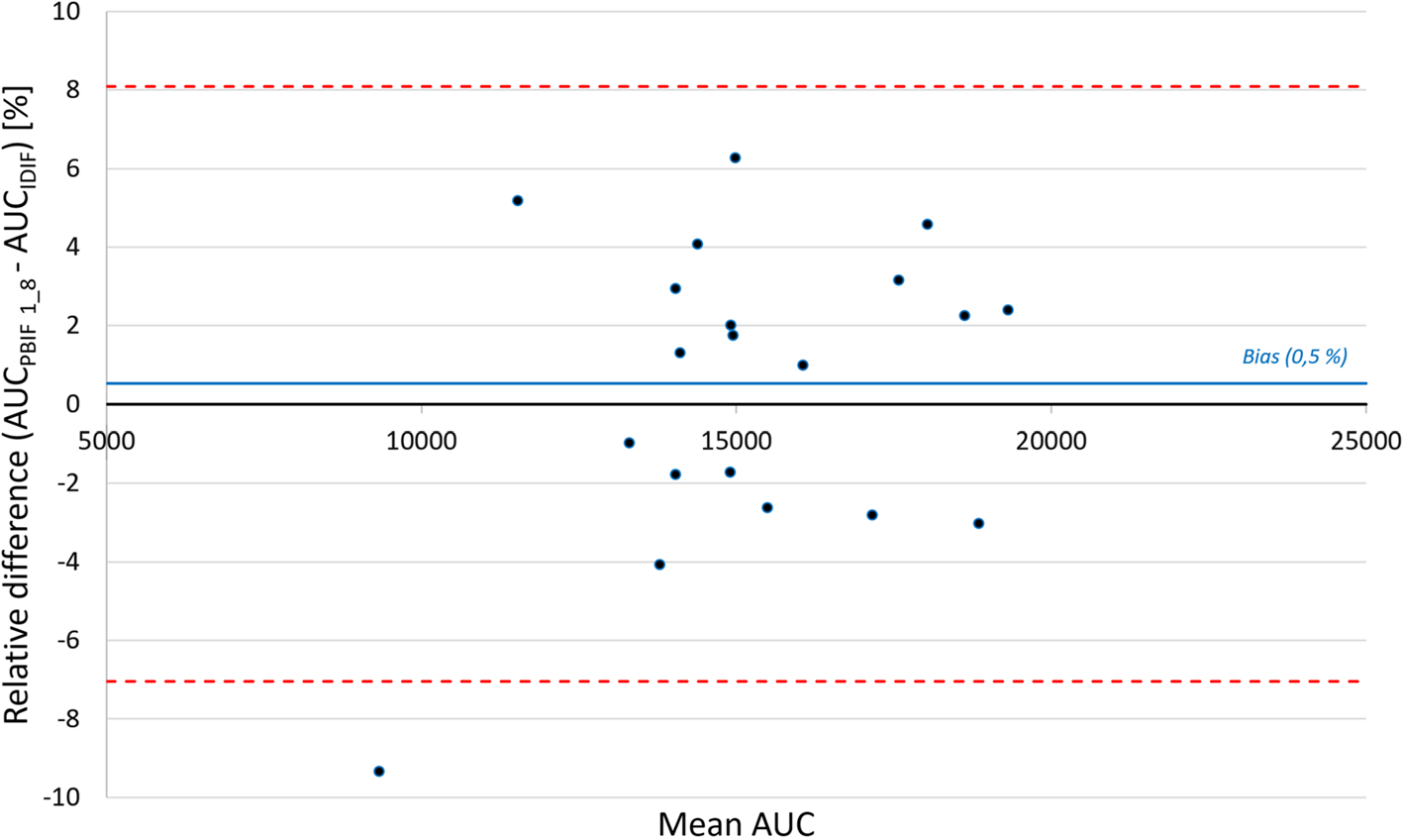
Bland–Altman plot of mean relative difference between AUC_PBIF_ and AUC_IDIF_ (scaling time 1_8) in each patient of the validation group G2 (*n* = 20). Blue solid line = mean bias; red dashed lines = IC95%

Figure 2 shows a Bland–Altman plot of mean difference between AUC_PBIF_ and AUC_IDIF_ using 1_8 passes scaling time in the validation group G2.

The mean ± SD relative bias was calculated at 0.5 ± 3.8%. The correlation coefficient (*R*^2^) between the two series was 0.961 (AUC_PBIF_ = 1.007 × AUC_IDIF_).

Table 3 shows errors on PBIF AUC depending on the time window used for normalization in our study and the literature. Due to the limitation of the soft, addressed in the discussion, our PBIF is normalized to the total number of points acquired (11–70 min). Naganawa et al. compared the AUC errors between PBIF normalized over two different time windows relative to AIF. Dias et al. also showed the influence of other pathologies, such as the diabetes, on the results.

**Table 3:** Comparison with literature on errors of PBIF AUC regarding on scaling times

Scaling times	Our study (relative to IDIF)	Naganawa et al. [28] (relative to AIF)		Dias et al. [32] (relative to AIF)	
				Diabetes no	Diabetes yes
	11–70 min	15–45 min	30–60 min	50–70 min	50–70 min
R^2^	0.96	0.93	0.94	0.93	0.83
Bias	0.5%	-1%	3%	2%	7%
SD	4%	6%	6%	4%	6%

### Parametric analysis

#### Optimal time window selection

A total of 44 lesions of metastatic melanoma (MM) were identified in the validation group 2. Results of statistical correlation (*R*^2^, mean bias ± SD) between both Ki mean and Ki max values obtained with PBIF and IDIF on MM lesions depending on each studied time windows are shown in Tables 4 and 5. The corresponding intercept values (Vb) are given in Additional file 2: Tables S1 and S2.

**Table 4:** Results of statistical correlation (R2, bias and SD) between mean Ki values of 44 MM lesions using PBIF and IDIF depending on different time windows

N = 44	2_4	2_5	2_7	3_5	3_6	4_6	4_7	5_7	5_8
R^2^	0.998	0.998	0.996	**0.999**	0.997	0.998	0.998	**0.999**	0.995
Bias	− 8.1%	− 9.3%	− 9.3%	-5.4%	− 6.0%	− 4.9%	− 6.2%	− 5.2%	− **4.9%**
SD	7.4%	8.7%	6.8%	5.2%	8.1%	6.8%	7.4%	**4.1%**	6.5%

**Table 5:** Results of statistical correlation (R2, bias and SD) between maximal Ki values of 44 MM lesions using PBIF and IDIF depending on different time windows

**N = 44**	2_4	2_5	2_7	3_5	3_6	4_6	4_7	5_7	5_8
R^2^	0.998	**0.999**	0.998	**0.999**	0.997	0.999	0.998	**0.999**	0.997
Bias	− 8.2%	− 8.3%	− 7.1%	− 5.2%	− 5.4%	− 5.8%	− 6.4%	− 5.7%	− **4.8%**
SD	6.3%	6.4%	4.6%	5.4%	6.8%	4.6%	5.3%	**3.6%**	5.6%

All R^2^ were higher than 0.995 with mean bias lower than 10%. Mean bias ± SD tended to decrease within post-injection time. Results were comparable using both Ki mean and Ki max.

In combining statistical results and proximity to usual static PET acquisition of routine practice, optimal 3 passes and 4 passes time windows were, respectively, 5_7 (*R*^2^ = 0.999 for both Ki mean and Ki max) and 5_8 (mean ± SD bias = − 4.9 ± 6.5% for Ki mean and − 4.8% ± 5.6% for Ki max), most relevant results are in bold.

**Fig. 3:**
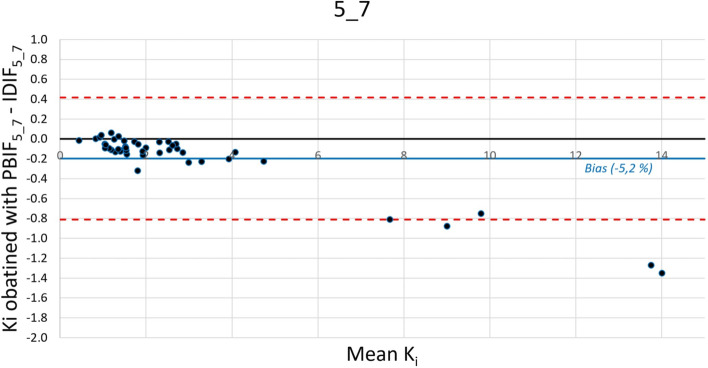
Bland–Altman plot of mean K_i_ values (100*ml/ml/min) of 44 tumoral lesions obtained with PBIF_5_7_ and IDIF_5_7_. Blue solid line = mean bias; red dashed lines = IC95%

**Fig. 4:**
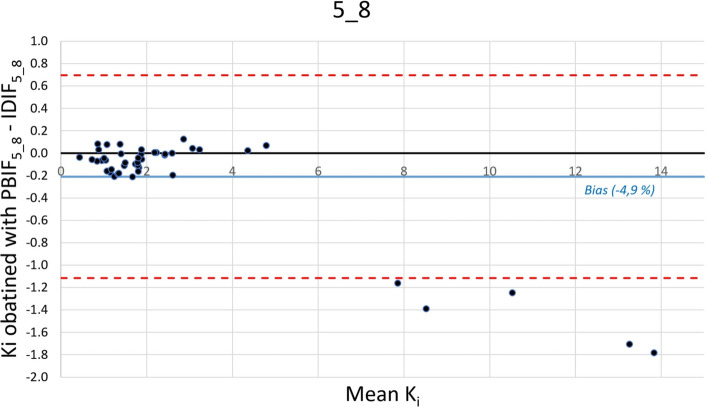
Bland–Altman plot of mean Ki values (100*ml/ml/min) of 44 tumoral lesions obtained with PBIF_5_8_ and IDIF_5_8_. Blue solid line = mean bias; red dashed lines = IC95%

Figures 3 and 4 show a Bland–Altman plot of error on Ki generated by PBIF compared to IDIF using 5_7 and 5_8 time window. Variations are less important using 3 passes against 4, as shown as the comparison of the two Bland–Altman plot with the increase of the limit of agreement passing from 5_7 to 5_8 passes.

#### Validation on pathological and physiological uptakes

**Fig. 5:**
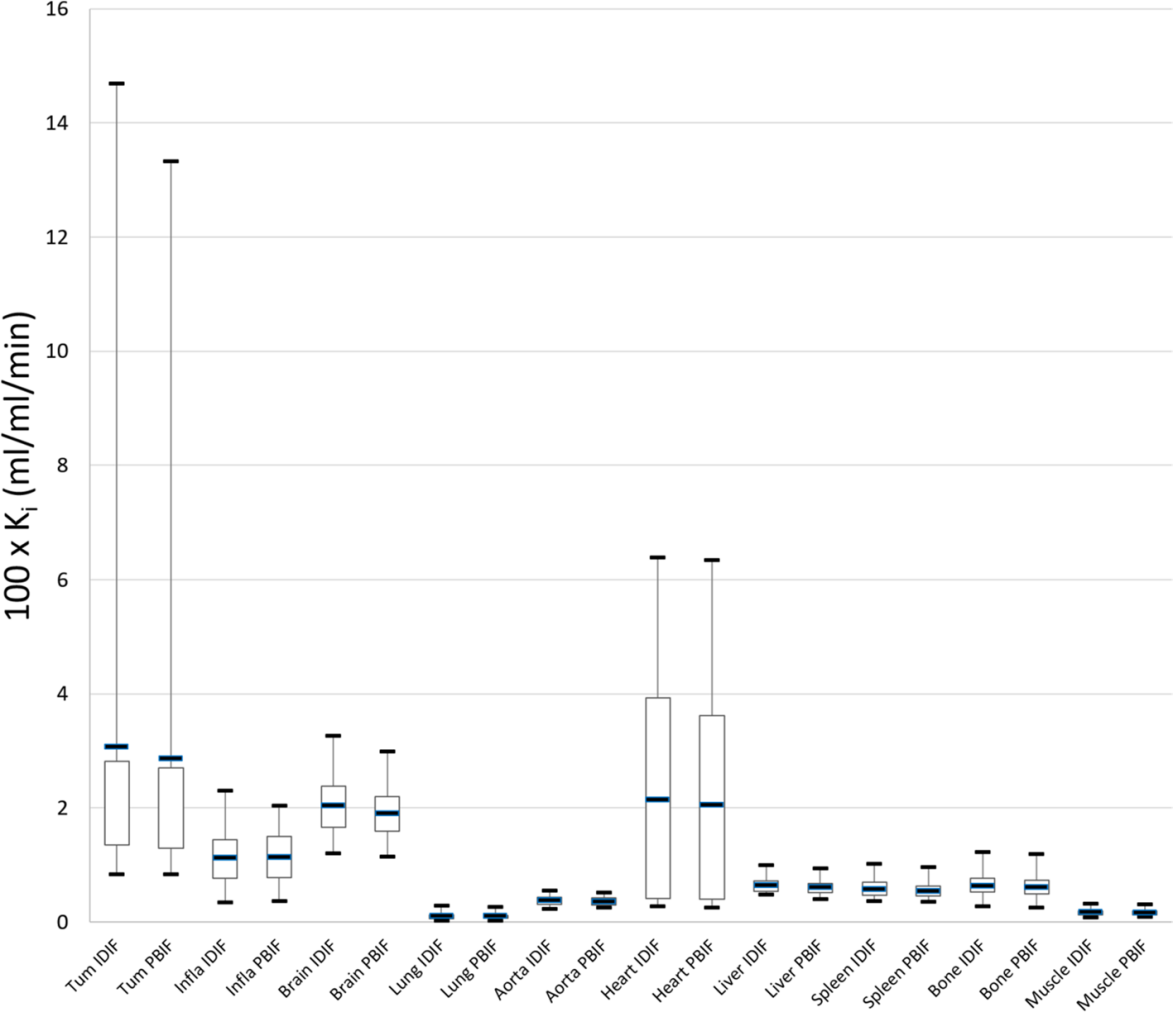
Box plots of Ki mean values obtained with IDIF_5_7_ and PBIF_5_7_ for pathological (tumor (Tum), inflammation (Infla)) and physiological uptakes

The difference between Ki values of pathological (tumor, inflammation) and physiological uptakes using both PBIF and IDIF with the 5_7 time window is presented in Table 6 and shown on the boxplot Fig. 5. The corresponding values for the intercept (Vb) are given in Additional file 3: Table S3. There was no significant difference in Ki values using both PBIF and IDIF (*p* > 0.05).

**Table 6:** Comparison between mean [range] Ki mean values (100ml/ml/min) obtained with IDIF_5_7_ and PBIF_5_7_ for pathological (tumor, inflammation) and physiological uptakes

	K_i_ (IDIF_5_7_)	K_i_ (PBIF_5_7_)	p value
Tumor (*n* = 44)	3.07 [0.84; 14.69]	2.87 [0.83; 13.33]	0.586
Immune induced inflammation (*n* = 11)	1.13 [0.35; 2.30]	1.13 [0.37; 2.04]	0.748
Brain (n = 20)	2.04 [1.21; 3.26]	1.90 [1.15; 2.99]	0.49
Lung (n = 20)	0.11 [0.03; 0.29]	0.10 [0.02; 0.26]	0.81
Aorta (n = 20)	0.38 [0.23; 0.55]	0.36 [0.26; 0.52]	0.59
Heart (n = 20)	2.15 [0.27; 6.39]	2.05 [0.25; 6.34]	0.72
Liver (n = 20)	0.64 [0.49; 1.00]	0.61 [0.41; 0.94]	0.22
Spleen (n = 20)	0.58 [0.37; 1.03]	0.54 [0.35; 0.97]	0.59
Bone (n = 20)	0.63 [0.28; 1.23]	0.61 [0.25; 1.20]	0.54
Muscle (n = 20)	0.17 [0.09; 0.32]	0.17 [0.09; 0.31]	0.51

On tumor lesions analysis (44 melanoma metastasis), there was no significant difference in mean ± SD K_i_ (IDIF_5_7_) and K_i_ (PBIF_5_7_) (3.07 ± 3.27 and 2.86 ± 2.96 100ml/ml/min, *p* = 0.586). Additional file 1: Figure S1 shows the comparison of a Ki axial slice reconstructed from an IDIF and a PBIF over the 5_7 time window, and the corresponding SUV image.

On inflammatory disease analysis (11 pseudo-progressive lesions), there was no significant difference in mean ± SD K_i_ (IDIF_5_7_) and K_i_ (PBIF_5_7_) (1.13 ± 0.59 and 1.13 ± 0.55 100ml/ml/min, *p* = 0.98).

On physiological uptake analysis, there was no significant difference in K_i_ (IDIF_5_7_) and K_i_ (PBIF_5_7_) values whatever the considered organ (*p* > 0.05). The highest dispersion in Ki values was found for the myocardium (range of 0.61 to 8.88 and of 0.54 to 8.55 for K_i_ (IDIF_5_7_) and K_i_ (PBIF_5_7_), respectively).

## Discussion

Dynamic whole-body PET (dWB-PET) imaging provides additional information to usual static SUV metrics, particularly on the tracer influx rate (Ki) through pathological or physiological tissues [13]. Knowledge of spatio-temporal distribution of radiotracer could allow to better differentiate tumor lesions from inflammation and thus influence the response assessment to therapy in oncology. Although the principle of multiparametric PET imaging has been described for several decades, its use in clinical routine has only become feasible in rare nuclear medicine departments, equipped with specific PET systems integrating automated workflows. Indeed, such workflows are simpler and less invasive than arterial blood sampling, in using an image-derived input function (IDIF) to quantify blood activity concentration through acquisition time but are still time-consuming. Nevertheless, optimizations are still needed to make dWB-PET imaging acceptable in daily practice. The recent proposal for population-based IF (PBIF) modeling is an area of research allowing to significantly reduce imaging time and improve patient comfort [31–33].

Our study shows the clinical feasibility of a short duration WB dynamic 18FDG-PET imaging using a PBIF without significant differences in Ki quantification, in a cohort of patients with metastatic melanoma treated by immunotherapy.

### PBIF creation process

In our study, the PBIF model was created from a cohort of 17 controlled patients, a sufficient sample size according to published data (varying from 11 to 23) [28, 40]. The low mean relative bias (0.5%) between the AUCs of the scaled PBIF and the IDIFs of the MM cohort shows that a model created from healthy patients can be applied to a specific pathology.

We choose to collect for each case an IDIF using an automatically defined volume of interest (VOI) in the left ventricle. In a previous study of 24 patients, Sari et al. [41] found a good agreement between the amplitudes of the peaks and tails of IDIFs derived from ascending aorta, descending aorta, left ventricle, and left atrium. Most importantly, they emphasized that an IDIF measured from the carotid arteries underestimated the AUC due to uncorrected partial volume effects.

After resampling of different TACs, we synchronize IDIF on the shortest time-to-peaks, unlike Sari et al. [33] who used the mean time-to-peak. The use of the shortest time to peak could be one of the causes of the negative bias observed when using PBIF. Indeed, when using the model, the real time-to-peak information cannot be taken into account. The impact of such difference on our overall results will be assessed in a further comparative study.

We fitted the average IDIF according to the Feng model. This mathematical function was the most used in the recent literature, as example by Naganawa et al., Dias et al. and Sari et al. [28, 33, 42]. Like their results, we found an excellent correlation between the measurements and the model (*R*^2^ = 0.998). Our maximum shape deviation was observed only in the first 20 s post-injection and was quantified at an insignificant fraction of 0.5% of the total AUC. This result was comparable to those reported in the respective series of Naganawa et al. [28] (− 1 ± 6% for a 15–45-min scaling time window, and 3 ± 6% for a 30–60 scaling time window) and Dias et al. [32] (− 3 ± 6% for a 30–50 scaling time window; − 2 ± 7% for a 40–60 scaling time window; both with Feng model but compared to arterial IF). The fit of the input function is an important factor in the quality of the results. Overfitting or underfitting can lead to over- or underestimation of Ki. Studies have shown that an error of 20% on the AUC of the IF leads to a deviation of around 4% on the Ki [29, 43]. Other models can be used to describe IF. We used a sum of a gamma variate function with three-exponential method, while Dias et al. [32] also compared with a four-exponential model with good results (2% to 0% bias, respectively, for a 40–60 scaling time window) but comparing PBIF to AIF.

### PBIF and Ki comparisons

One of the parameters that will influence parametric reconstruction is the time window used to scale the PBIF, especially when using the latest points [28]. In our study, PBIF was scaled using the whole late dynamic acquisition (i.e., 11–70 min). It would have been of interest to try multiple scaling windows as already assessed in the literature [31, 41]. Nevertheless, this was not possible in our study due to the software limitations of our PET system. However, this concern does not appear to be crucial for the PBIF scaling. Indeed, Sari et al. [33] evaluated different scaling windows and concluded that the use of a late window (i.e., 55–65 min) would provide the lowest bias in their study. Thus, our results remain valid because the use of a late scaling window, concomitant with the window used for the Patlak analysis, will provide a similar accuracy.

Once the IF has been reconstructed, our method of testing 9 different time windows (TWs) of dWB-PET data (3 or 4 passes at 15- to 70-min post-injection time) to compare scaled PBIF and measured IDIF on the respective Patlak reconstructions is consistent with the literature [28, 31, 32]. In our results, the TW used to perform the direct 4D nested Patlak reconstruction affected the Ki measurements, even if the bias was overall acceptable (less than 10% regardless of the time window). Indeed, the Ki bias was lower in late time windows (around 5% for 35–53 min, 45–61 min, 45–70 min time windows) than in early time windows (8% to 9% for 20–35 min, 20–45 min, 20–61 min time windows).

Our best selected reconstructions in terms of optimal use in routine practice and statistical results were 45–61 min and 45–70 min TWs. Indeed, we showed a very good correlation between IDIF and PBIF reconstructions (bias − 5.2% and − 4.9%, SD 4.1% and 6.5%, *R*^2^ 0.999 and 0.997, respectively). We observed a slight underestimating of Ki metrics, especially in case of high value as already described [44]. Effectively the 5 highest Ki mean values showing higher differences between IDIF_5_7_ and PBIF_5_7_ (− 0.8 to − 1.4 absolute difference, − 7 to − 15%) were measured on the same patient showing very high tumor uptake on standard PET images (SUV values around 100).

Surprisingly, we found a negative bias, although it has been described between + 1.5 [33] to + 7.4% [31] and even up to + 23% [31] but with a very short 10-min acquisition. It can also be a PET system effect. Indeed, the positive bias occurs on large FOV PET system, while on standard FOV the bias remains negative [28, 32]. In any cases, the bias generated by our model remained lower than the difference between pathological and physiological Ki values. These results are promising, as a 20 min of WBdyn PET acquisition time in daily practice remains largely acceptable for both the patient’s compliance and the department’s organization.

### Validation on pathological and physiological uptakes

We chose to use a VOI predefined on the gold-standard 3D SUV image for Ki metrics extraction of each pathological lesion or physiological organ in the IDIF and PBIF parametric images. Although parametric PET images are known to show a better contrast and attain superior quantification, they are also typically more sensitive to noise [37] and may suffer from kinetic artifacts compared to static SUV images. Indeed, SUV images use projection acquired 1h post-injection (PI), while 2_4 Patlak reconstruction use projection acquired during the 20–35 min PI for example. Consequently, we decided to measure both mean and maximum Ki metrics in VOIs because mean values are less sensitive to noise, while maximum values are less sensitive to kinetic blur. Ultimately, both measurements showed similar bias (difference lower than 1%) and correlations when comparing IDIF and PBIF-based reconstructions, regardless of the chosen time window.

We found no significant difference in Ki values of both tumoral lesion, inflammatory disease, and physiological uptakes in comparing IDIF_5_7_ and PBIF_5_7_. Regarding physiological Ki values, our results were comparable with literature data [42, 45]. As Dias et al., we found widest variation was found on myocardial Ki values (range from 0.54 to 8.55 100ml/ml/min with PBIF_5_7_), probably explained by the absence of specific cardiac free fatty-acid consumption diet to suppress physiological myocardial uptake. Regarding tumoral lesions, no specific published data on melanoma are available for comparison. Sari et al. [45] only described 2 lesions of melanoma with mean Ki of 1.3 and 3.9 100ml/ml/min (average of 2.87 100ml/ml/min [0.83; 13.33] in our results). These findings were globally concordant, regarding Vd values, in terms of bias, less than 5%, for each time window. However, the dispersion is higher than for Ki, with SD ranging from 6.4 to 22.1%.

To the best of our knowledge, this is the first study applying PBIF approach trying to resolve a clinical issue in routine practice, in applying our model to a cohort of 20 patients with metastatic melanoma (MM) treated by immunotherapy (ICI). In these preliminary results of the IMMUNOPET2 study, we found that 4D whole-body dynamic PET images might be capable to differentiate progression disease (PD) to pseudo-progression (PP) (mean Ki values 2.87 [0.83–13.33] versus 1.13 [0.37–2.04]). This capability of Ki values to differentiate malignant to inflammatory lesions has recently been suggested by Shawran et al. [21], but in a large selection of tumor type and inflammations etiologies.

### Perspectives

As a perspective, another axis of optimization would be to assess the impact of reconstruction parameters on Ki measurements. Indeed, Patlak imaging is sensitive to noise, though the impact is relatively less for direct 4D nested Patlak reconstructions. This would allow us to evaluate the reproducibility of the Ki measurements and to establish the best ratio between detectability and image quality. Wu et al. [37] have already published a study with this approach by applying a denoising filter during reconstructions. In a cohort of 65 patients, they proved a similar detectability of lesions by reducing acquisition time by two. Finally, a more efficient and less complex approach to manage image noise in dynamic WB PET data would be to exploit high sensitivity scanners, such as large axial FOV or total-body PET systems [31, 33, 37]. Although this type of device is not yet widely available from manufacturers, is currently expensive and thus not widely adoptable in clinic [46, 47], it will definitely open a new era in parametric PET imaging.

## Conclusion

This article highlights the methodology for obtaining direct voxelwise parametric imaging. We showed the feasibility of shortened whole-body dynamic 18FDG-PET protocols and respective whole-body Patlak Ki imaging using a population-based input function on a Vision 600 PET/CT system. We demonstrated that using such PBIF allows reducing dWB-PET acquisition duration from 70 to 20 min with reasonable bias. These findings open perspectives for the adoption of dWB-PET in clinical routine for a wide range of indications, including treatment response in oncology.

### Supplementary Information

**Additional file 1**: Fig. S1 Reconstructed Ki image with an IDIF5_7 (a) and a PBIF5_7 (b). In comparison, SUV image (c) shows a lower tumor-to-normal tissue ratio.

**Additional file 2**: Table S1 Results of statistical correlation (R2, bias and SD) between maximal Vd values of 44 MM lesions using PBIF and IDIF depending on different time windows. Table S2 Results of statistical correlation (R2, bias and SD) between maximal Vd values of 44 MM lesions using PBIF and IDIF depending on different time windows.

**Additional file 3**: Table S3 Comparison between mean [range] Vd mean values (100ml/ml/min) obtained with IDIF5_7 and PBIF5_7 for pathological (tumor, inflammation) and physiological uptakes.

## Data Availability

The datasets used and/or analyzed during the current study are available from the corresponding author on reasonable request.
