# Supplementary material for: Clinical application of a population-based input function (PBIF) for a shortened dynamic whole-body FDG-PET/CT protocol in patients with metastatic melanoma treated by immunotherapy
Source: EJNMMI Phys. 2023 Dec 8;10:79. doi: 10.1186/s40658-023-00601-3 (PMC10703763; doi:10.1186/s40658-023-00601-3)
Supplement: Supplementary file 1 — Additional file 1: Fig. S1 Reconstructed Ki image with an IDIF5_7 (a) and a PBIF5_7 (b). In comparison, SUV image (c) shows a lower tumor-to-normal tissue ratio. [file 40658_2023_601_MOESM1_ESM.pdf]

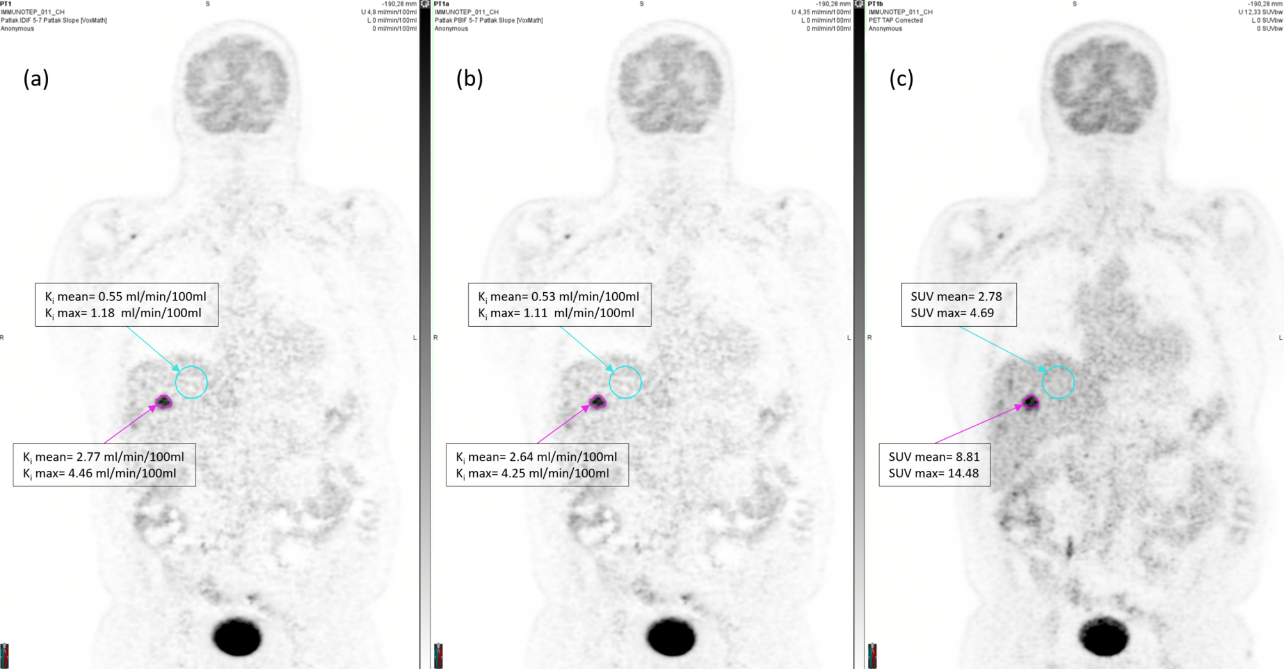

PT1
IMMUNOTEP_011_CH
Patlak IDIF 5-7 Patlak Slope [VoxMath]
Anonymous
-190,28 mm
U 4,8 ml/min/100ml
L 0 ml/min/100ml
0 ml/min/100ml
(a)
K_i mean= 0.55 ml/min/100ml
K_i max= 1.18 ml/min/100ml
K_i mean= 2.77 ml/min/100ml
K_i max= 4.46 ml/min/100ml
PT1a
IMMUNOTEP_011_CH
Patlak PBIF 5-7 Patlak Slope [VoxMath]
Anonymous
-190,28 mm
U 4,35 ml/min/100ml
L 0 ml/min/100ml
0 ml/min/100ml
(b)
K_i mean= 0.53 ml/min/100ml
K_i max= 1.11 ml/min/100ml
K_i mean= 2.64 ml/min/100ml
K_i max= 4.25 ml/min/100ml
PT1b
IMMUNOTEP_011_CH
PET TAP Corrected
Anonymous
-190,28 mm
U 12,33 SUVbw
L 0 SUVbw
0 SUVbw
(c)
SUV mean= 2.78
SUV max= 4.69
SUV mean= 8.81
SUV max= 14.48
