# Supplementary material for: Clinical application of a population-based input function (PBIF) for a shortened dynamic whole-body FDG-PET/CT protocol in patients with metastatic melanoma treated by immunotherapy
Source: EJNMMI Phys. 2023 Dec 8;10:79. doi: 10.1186/s40658-023-00601-3 (PMC10703763; doi:10.1186/s40658-023-00601-3)
Supplement: Supplementary file 2 — Additional file 2: Table S1 Results of statistical correlation (R2, bias and SD) between maximal Vd values of 44 MM lesions using PBIF and IDIF depending on different time windows. Table S2 Results of statistical correlation (R2, bias and SD) between maximal Vd values of 44 MM lesions using PBIF and IDIF depending on different time windows. [file 40658_2023_601_MOESM2_ESM.docx]

| N=44 | 2_4 | 2_5 | 2_7 | 3_5 | 3_6 | 4_6 | 4_7 | 5_7 | 5_8 |
| --- | --- | --- | --- | --- | --- | --- | --- | --- | --- |
| R² | 0.998 | 0.998 | 0.996 | 0.999 | 0.997 | 0.998 | 0.998 | 0.999 | 0.995 |
| Bias | -0.6% | 1.71% | 4.67% | -1.1% | 4.2% | -1.6% | 2.5% | -0.9% | -3.6% |
| SD | 15.7% | 15.0% | 11.9% | 8.8% | 12.3% | 6.4% | 9.8% | 11.9% | 22.1% |

Table S1 Results of statistical correlation (R2, bias and SD) between mean Vd values of 44 MM lesions using PBIF and IDIF depending on different time windows.

| N=44 | 2_4 | 2_5 | 2_7 | 3_5 | 3_6 | 4_6 | 4_7 | 5_7 | 5_8 |
| --- | --- | --- | --- | --- | --- | --- | --- | --- | --- |
| R² | 0.998 | 0.999 | 0.998 | 0.999 | 0.997 | 0.999 | 0.998 | 0.999 | 0.997 |
| Bias | -0.35% | -4.77% | -4.77% | -2.3% | -1.72% | -3.18% | -0.81% | -4.23% | -1.65% |
| SD | 10.3% | 29.8% | 9.8% | 7.3% | 7.8% | 3.9% | 5.8% | 6.67% | 4.3% |

Table S2 Results of statistical correlation (R2, bias and SD) between maximal Vd values of 44 MM lesions using PBIF and IDIF depending on different time windows.
