# Supplementary material for: Clinical application of a population-based input function (PBIF) for a shortened dynamic whole-body FDG-PET/CT protocol in patients with metastatic melanoma treated by immunotherapy
Source: EJNMMI Phys. 2023 Dec 8;10:79. doi: 10.1186/s40658-023-00601-3 (PMC10703763; doi:10.1186/s40658-023-00601-3)
Supplement: Supplementary file 3 — Additional file 3: Table S3 Comparison between mean [range] Vd mean values (100ml/ml/min) obtained with IDIF5_7 and PBIF5_7 for pathological (tumor, inflammation) and physiological uptakes. [file 40658_2023_601_MOESM3_ESM.docx]

|  | V_d_ (IDIF_5_7_) | V_d_ (PBIF_5_7_) | p value |
| --- | --- | --- | --- |
| Tumor (n=44) | 107.68 [10.61; 459.75] | 105.11 [11.60; 135.93] | 0.865 |
| Immune induced inflammation (n=11) | 61.76 [31.44; 135.93 ] | 64.71 [33.49; 131.51] | 0.401 |
| Brain (n=20) | 66.66 [39.95; 161.39] | 65.95 [35.84; 178.35] | 0.402 |
| Lung (n=20) | 11.48 [5.68; 19.42] | 10.93 [5.30; 19.10] | 0.667 |
| Aorta (n=20) | 60.54 [46.37; 70.97] | 57.55 [46.35; 65.37] | 0.086 |
| Heart (n=20) | 83.27 [39.48; 275.29] | 81.11 [38.16; 263.24] | 0.838 |
| Liver (n=20) | 59.80 [40.08; 73.72] | 57.34 [43.08; 69.16] | 0.402 |
| Spleen (n=20) | 47.67 [34.92; 61.79] | 45.84 [32.01; 68.11] | 0.381 |
| Bone (n=20) | 31.76 [6.31; 70.27] | 30.32 [5.03; 66.51] | 0.897 |
| Muscle (n=20) | 11.32 [5.77; 18.63] | 10.87 [5.36; 17.17] | 0.697 |

Table S3 Comparison between mean [range] Vd mean values (100ml/ml/min-1) obtained with IDIF_5_7_ and PBIF_5_7_ for pathological (tumor, inflammation) and physiological uptakes.
